# Effects of Lead and Mercury on Sulfate-Reducing Bacterial Activity in a Biological Process for Flue Gas Desulfurization Wastewater Treatment

**DOI:** 10.1038/srep30455

**Published:** 2016-07-26

**Authors:** Liang Zhang, Xiaojuan Lin, Jinting Wang, Feng Jiang, Li Wei, Guanghao Chen, Xiaodi Hao

**Affiliations:** 1 School of Chemistry & Environment, South China Normal University, Guangzhou, China; 2 SYSU-HKUST Research Center for Innovation Environmental Technology, Sun Yat-sen University, Guangzhou, China; 3 Key Laboratory of Theoretical Chemistry of Environment, Ministry of Education, Guangzhou, China; 4 Department of Civil & Environmental Engineering, The Hong Kong University of Science and Technology, Clear Water, Kowloon, Hong Kong, China; 5 Key Laboratory of Urban Stormwater System and Water Environment -MoU/R and D Center for Sustainable Wastewater Treatment, Beijing University of Civil Engineering and Architecture, Beijing, China

## Abstract

Biological sulfate-reducing bacteria (SRB) may be effective in removing toxic lead and mercury ions (Pb(II) and Hg(II)) from wet flue gas desulfurization (FGD) wastewater through anaerobic sulfite reduction. To confirm this hypothesis, a sulfite-reducing up-flow anaerobic sludge blanket reactor was set up to treat FGD wastewater at metal loading rates of 9.2 g/m^3^-d Pb(II) and 2.6 g/m^3^-d Hg(II) for 50 days. The reactor removed 72.5 ± 7% of sulfite and greater than 99.5% of both Hg(II) and Pb(II). Most of the removed lead and mercury were deposited in the sludge as HgS and PbS. The contribution of cell adsorption and organic binding to Pb(II) and Hg(II) removal was 20.0 ± 0.1% and 1.8 ± 1.0%, respectively. The different bioavailable concentration levels of lead and mercury resulted in different levels of lethal toxicity. Cell viability analysis revealed that Hg(II) was less toxic than Pb(II) to the sludge microorganisms. In the batch tests, increasing the Hg(II) feeding concentration increased sulfite reduction rates. In conclusion, a sulfite-reducing reactor can efficiently remove sulfite, Pb(II) and Hg(II) from FGD wastewater.

Mercury and lead emissions from coal combustion power plants is a serious environmental issue because of their detrimental impacts on human health and ecological system^1^,^2^,^3^. Yan *et al*. reported that SO_2_ emissions from Chinese electric power plants were 913 million tons in 2012^4^. The wet flue gas desulfurization (FGD) process, a commonly applied technology for the removal of SO_2_ from flue gas, can also remove some heavy metals^5^; thus, the generation of large amounts of FGD wastewater can also contain various heavy metals, (e.g., arsenic, lead, mercury and selenium)^5^,^6^. Among these heavy metals, lead and mercury are the two most common toxic metals that need to be further removed before wastewater discharge^5^,^6^. Generally, FGD wastewater is treated with hydroxide and sulfide salts to remove Pb(II) and Hg(II)^6^ and to meet strict discharge limits, e.g., 10 μg/L Hg and 100 μg/L Pb in the United States^2^,^3^ and 50 μg/L Hg and 1 mg/L Pb in China^7^.

The limestone wet FGD process is the most popular process for desulfurization in coal-burning power plants, especially in China^4^. However, this process generates large amounts of gypsum-containing waste and often causes blockages at facilities because of calcium sulfate deposits^8^. Alternatively, the biological treatment of FGD wastewater via sulfate-reducing bacteria (SRB) is attractive because SRB can simultaneously remove sulfate/sulfite, nitrate/nitrite, heavy metals and organic matter with a low sludge yield if electron donors are supplied^9^,^10^,^11^. For instance, Bio-FGD (biological FGD), a typical biological sulfate reduction process initiated by Paque, has been adopted by a power plant in China using citrate wastewater as the carbon source^12^. Because sulfide is generated by SRB under anaerobic conditions, the removal of Hg(II) and Pb(II) in this process is expected to occur as described in Equations 1 9,12,13 (taking acetate as the typical electron donor here):


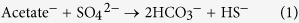



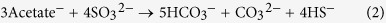



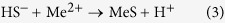


However, mercury and lead are known to interfere with important microbial processes, including the aerobic and anaerobic degradation of organic matters^14^,^15^. Their toxic effects include ion displacement and/or substitution of essential ions from cellular sites and blockade of essential functional groups, for example, enzymes, polynucleotides, and essential nutrient transport systems. The toxic effects of mercury and lead result in modifications of the active conformation of biological molecules (including denaturation and inactivation of enzymes) and disruption of the integrity of cellular and organelle membranes^16^,^17^. SRB also play a key role in the formation of methylmercury (MeHg), which is the most poisonous form of mercury, in the presence of organic compounds and sulfate^18^. Acute toxicity, reported as lethal concentration 50 (LC50) values of MeHg, ranged from 24 μg/L to 54 μg/L^19^,^20^. For this reason, the treatment of mercury-contaminated wastewater by SRB is limited^21^. The toxicities imply that the existence of Hg(II) and Pb(II) in FGD wastewater may be detrimental to SRB and lead to the instability or decline in performance in a biological FGD wastewater treatment process. The adverse effects from heavy metals would be a challenge to biological FGD wastewater treatment processes, but so far, it has not been well investigated.

Furthermore, the effects of mercury and lead on the activity of SRB are different and even contradictory. Haritha *et al*. reported that hypersaline SRB activity was inhibited by Hg(II) but stimulated by Pb(II) under exposure to mercury or lead concentrations ranging from 50 to 500 mg/L^22^. However, Kumar Sani *et al*. found that the growth of *Desulfovibrio desulfuricans* was completely inhibited when exposed to 3 mg/L Pb(II)^23^. In addition, mercury and lead were found to stimulate the growth of SRB (*Desulfosarcina*) in the presence of acetate^24^. These findings reveal that different SRB species respond differently to mercury and lead exposure.

Hence, in a biological FGD wastewater treatment system, the effects of mercury and lead on the activity of SRB require investigation, especially the effects of the combined presence of both Pb(II) and Hg(II).

To achieve the above, a laboratory-scale sulfite-reducing up-flow anaerobic sludge blanket (SrUASB) reactor was established to simulate the FGD biological wastewater treatment process and investigate its long-term performance on the removal of sulfite, organic matters, Pb(II) and Hg(II). The changes in the SRB community were analyzed to determine the SRB genera that tolerate metals. Batch tests were also conducted to evaluate the activity of SRB at elevated concentrations of Pb(II) and Hg(II), including the sulfite reduction rate (SRR) and bacterial viability.

## Results and Discussion

### Performance of the SrUASB reactor

**Figure 1:**
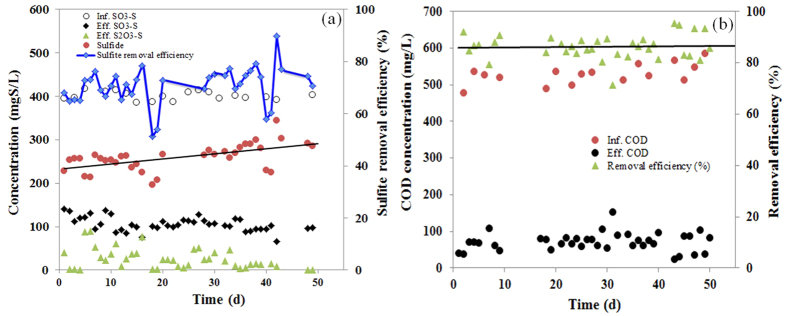
The performance of (**a**) sulfite reduction and sulfide production, and (**b**) organic matter removal in the SrUASB reactor.

The SrUASB reactor was operated for 50 days continuously to treat FGD wastewater, using domestic wastewater as the carbon source. The influent of the system consisted of 529 ± 50 mg/L chemical oxygen demand (COD), 386 ± 15 mg/L SO_3_^2−^-S, 32 mg/L NH_4_^+^-N, 2.3 mg/L Pb(II), and 570 μg/L Hg(II). The reactor performed well, removing 72.5 ± 7% of SO_3_^2−^ and 86.4 ± 5% of COD and producing 259 ± 30 mg/L sulfide in the effluent (Table 1). As Fig. 1 shows, the removal efficiencies of sulfite and organic matter were stable at the beginning and then increased slightly over time. These results were similar to those of the sulfite- and sulfate-reducing UASB reactors in the laboratory-scale flue gas desulfurization-sulfite reduction, autotrophic denitrification and nitrification-integrated (FGD-SANI) and SANI processes^9^,^25^ (Table 1). These results reveal that the activity of SRB was not affected by the feeding of Pb(II) and Hg(II) at the loading rates of 9.2 g/m^3^-d Pb(II) and 2.6 g/m^3^-d Hg(II), respectively.

Given the high sulfide production, the high removal efficiencies of both Hg(II) and Pb(II) were expected. As shown in Table 1, greater than 99.5% and 99.8% of Pb(II) and Hg(II), respectively, were removed in the SrUASB reactor. During the entire operational period, the concentration of Pb(II) in the effluent was always below the detection limit, and the concentration of Hg(II) in the effluent was as low as 1.1 ± 0.9 μg/L. These findings indicate that the SrUASB reactor is capable of simultaneously removing sulfite, organic matter, Pb(II), and Hg(II).

### The fates of the removed Pb(II) and Hg(II)

**Figure 2:**
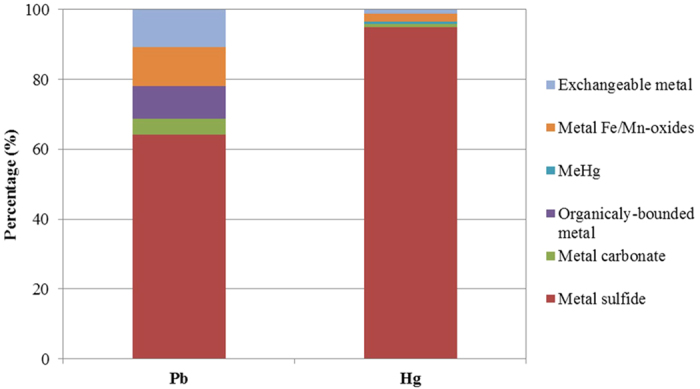
The fate of removed Pb(II) or Hg(II) deposited in the sludge of the SrUASB reactor.

Under anaerobic sulfite-reducing conditions, the potential pathways for the removal of Pb(II) and Hg(II) are metal sulfide precipitation, bio-sorption to cell surfaces^26^, intracellular penetration and accumulation^27^, capture, and detoxification by extracellular polymeric substances (EPS)^28^. Although Pb(II) and Hg(II) were effectively removed in the SrUASB reactor, the mechanisms by which the Pb(II) or Hg(II) are removed are unclear. By conducting batch tests, the species of lead and mercury that accumulated in the sludge were analyzed. As shown in Fig. 2, the removed Pb(II) and Hg(II) were mostly attributed to PbS and HgS formation, accounting for 64.3 ± 0.2% and 95.7 ± 11.1%, respectively. As expected, the precipitation of metal sulfide was the predominant process for the removal of Pb(II) and Hg(II) in the SrUASB reactor (Equation 3). This finding is consistent with those of Neculita *et al*.^29^. As Equations 1, 2 describe, dissimilatory sulfate/sulfite reduction produces bicarbonate as alkalinity and raises the pH value. Therefore, the pH in the effluent of the SrUASB reactor increased from 7.20 to 7.62, which is close to the optimum condition for the precipitation of PbS (pH 7.5–8.5)^26^ and HgS (pH < 8.0)^30^. The particles of metal sulfide formed served as biofilm carriers, resulting in the increase of the sludge retention inside the reactor^31^. In fact, the sludge concentration in the SrUASB reactor increased from 15.5 g VSS/L at the beginning of the experiment to 17.8 g VSS/L (volatile suspended solids) at the end of the experiment. This increase in sludge concentration could be the reason why the sulfide production rates gradually increased in the SrUASB reactor.

The exchangeable and organically bound metals represented the mercury and lead adsorbed and bound by cells or EPSs. In this study, cell adsorption and organic binding contributed 20.0 ± 0.1% to the removal of Pb(II) and 1.8 ± 1.0% to the removal of Hg(II) (Fig. 2). Considering that HgS had a much smaller K_sp_ (1 × 10^−54^) than that of PbS (1 × 10^−28^), the lower concentrations of mercury adsorbed and organically bound in the sludge are reasonable compared with that of Pb(II).

In addition, the results of the batch tests showed that 0.69% of the removed Hg(II) transformed into MeHg. MeHg was also observed in the sludge of the SrUASB reactor (Fig. S1 in Supplementary Information [SI]), and its concentration in the sludge at the end of the experiment was 0.29 ± 0.21 μg/g dry biomass. This result raised the concern that MeHg might accumulate in the sludge, although the methylation of mercury in the sulfite reduction process has not yet been thoroughly investigated. It can be inferred that the methylation of mercury is stimulated by the reduction of both sulfite and sulfate^32^. Nevertheless, the MeHg that accumulated in the sludge did not affect the activity of SRB in the 50-day laboratory-scale test.

### Effects of Pb(II) and Hg(II) on SRB activity

Under such low metal loading conditions, the performance of the SrUASB reactor did not seem to be influenced by either Hg(II) or Pb(II). However, when exposed to higher metal loading, the detrimental effects of Pb(II) and Hg(II) on the activity of SRB became apparent. To investigate the effects of Pb(II) and Hg(II) on the activity of SRB, batch tests were conducted under higher metal loading conditions. The sulfite reduction rate (SRR) was used as the indicator to reflect the activity of SRB.

**Figure 3:**
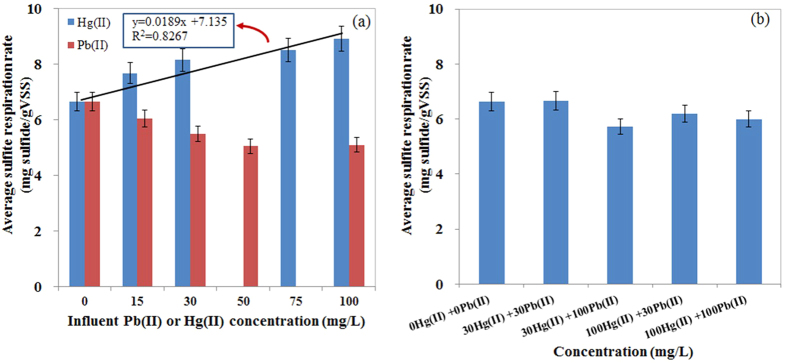
The effects of feeding Pb(II) or Hg(II) on the sulfite reduction rate: (**a**) individual feeding of Pb(II) or Hg(II), and (**b**) mixed feeding of Pb(II) and Hg(II).

The removal efficiencies of both Pb(II) and Hg(II) were consistently greater than 99.7% when the batch reactors were fed with wastewater containing 15–100 mg/L Pb(II) and Hg(II) for 96 hours (Table S1). However, the SRRs in relation to the feedings of Pb(II) and Hg(II) were different. Figure 3a shows that the SRRs decreased when Pb(II) concentrations increased from 0 to 100 mg/L. This finding agrees with that of Bharathi *et al*.^24^, who reported that concentrations of Pb(II) were responsible for a 50% growth inhibition of an isolated strain of SRB (*Desulfosarcina*). However, the effects of increasing Hg(II) concentrations on the sulfite reduction process showed a contradictory trend. The SRR was 8.90 mg sulfide/g VSS when SRB were exposed to 100 mg/L Hg(II), and it increased to 34% compared with the SRR in the control test (Fig. 3a). This result showed that Hg(II) promoted the SRR in the ranges from 0 to 100 mg/L. A linear relationship (R^2^ = 0.8267) was observed between the SRR and the spiked concentrations of Hg(II) in this study (Fig. 3a).

In these batch tests, the increased concentrations of Hg(II) stimulated the reduction of sulfite, a finding that is contradictory to previous studies. In general, the effect of mercury on SRB was as described by the Arndt-Schulz rule, which states that SRB are stimulated at low levels of mercury^22^,^33^,^34^ and inhibited at high concentrations of Hg(II) (100 and 200 mg/L)^22^,^24^. Nies *et al*. claimed that microorganisms do not benefit from Hg(II) because of its strong toxicity^35^. However, bacteria expressing the enzyme mercuric reductase (MerA), which is a very widespread enzyme in Hg-resistant bacteria, led to the detoxification of bacteria to Hg(II)^35^,^36^. In particular, the living cell can reduce Hg(II) to Hg^0^, which is subsequently released, bypassing the outer membrane, due to the extremely low redox potential of mercury and the vapor pressure/melting/boiling point of zero-valent mercury. Furthermore, the detoxification to Hg(II) is a mechanism requiring energy^36^. SRB must engage in higher activity to generate more energy. Therefore, the SRR increased when the concentrations of Hg(II) increased from 0 to 100 mg/L. The SRRs under the feeding condition of mixed heavy metals are shown in Fig. 3b. Increasing the feeding concentrations of Pb(II) and Hg(II) did not have significant impacts on the SRRs. This result indicates that sulfite reduction processes could be used to removed Pb(II)- and Hg(II)-contaminated wastewater even at high concentrations.

### Cell viability under Pb(II) and Hg(II) exposure

**Figure 4:**
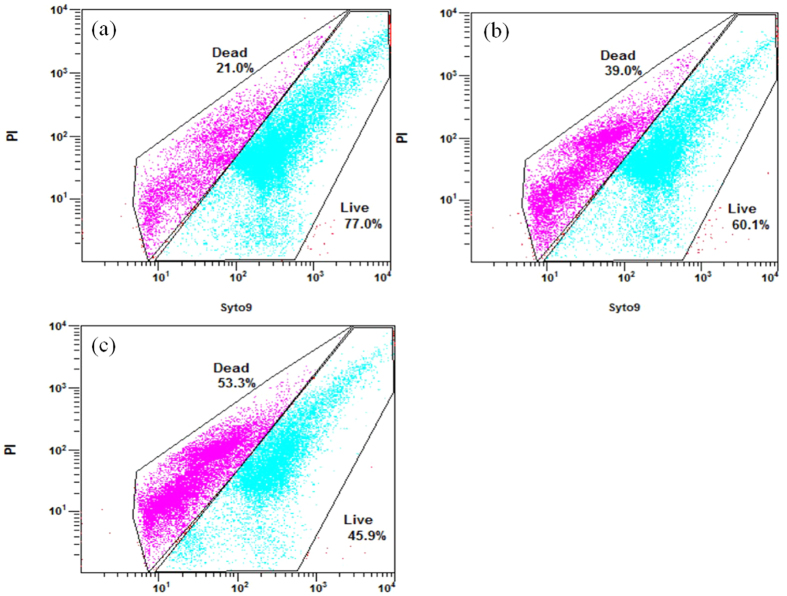
The proportions of live and dead cells in sludge samples incubated for 96 hours with (**a**) 100 mg Hg/L, (**b**) 100 mg Pb/L, and (**c**) metal-free wastewaters.

To validate the results of the batch tests, the cell viability of the sludge samples was analyzed using the LIVE/DEAD BacLight bacterial viability kit and then quantified using a flow cytometer. As Fig. 4 shows, more cells remained alive after 96 h of exposure to 100 mg/L Hg(II) than to 100 mg/L Pb(II). Cells exposed to 100 mg/L Hg(II) for 96 h experienced a death rate of 18.0%. By comparison, cells exposed to 100 mg/L Pb(II) for 96 h experienced a death rate of 32.3%.

Both Pb(II) and Hg(II) can penetrate the cell membrane to react with the intracellular components and replace the metal constituents in the active centers of the enzymes, cofactors or other biomolecules, resulting in the denaturation and inactivation of the enzymes and the disruption of the integrity of the cell organelle-membrane^16^,^17^,^37^. However, the toxicity of heavy metal to microbes depends on its bioavailability^38^. As presented in Fig. 2, the proportions of cell-adsorbed mercury and organically bound mercury were much smaller than for lead, implying that the bioavailability of mercury was lower than lead. This difference in bioavailability could be a reason why Hg(II) led to lower toxicity than Pb(II) in this study.

### Microbial community analysis

Because Pb(II) and Hg(II) affected the activity of SRB, these compounds could change the SRB community in the SrUASB reactor. Therefore, in the laboratory-scale test, sludge samples were taken from the SrUASB at days 1 and 50 for 16S rRNA gene sequencing to analyze the bacterial community profiles.

Approximately 11440 and 13784 raw pyrosequencing reads of the 16S rRNA gene were obtained from the sludge samples on the 1^st^ day (G1) and the 50^th^ day (G2). After filtering, 10172 and 11862 valid reads were analyzed by 16S rRNA pyrosequencing analysis (Table S3). Rarefaction curves based on the OTUs at 3% dissimilarity (Fig. S2) indicated that the sequences were sufficient to reflect the diversity of the microbial communities.

Altogether, 26 bacterial phyla were recovered from the sludge samples of the SrUASB reactor. For the seeding sludge, approximately 96% of the 16S rRNA gene sequences were affiliated with Chloroflexi, Firmicutes, Proteobacteria, Planctomycetes, Armatimonadetes, Actinobacteria and Synergistetes, representing approximately 35.5%, 30.5%, 11.2%, 9.2%, 4.3%, 2.8%, and 2.5% of the bacterial reads, respectively (Fig. S3a). After 50 days of operation, Chloroflexi, Firmicutes and Proteobacteria were still the main phyla in the SrUASB reactor. The relative abundance of Chloroflexi and Proteobacteria in the total 16S rRNA gene sequences increased slightly from 35.5 to 45.1% and from 11.2 to 12.8%, respectively. However, a decrease of Firmicutes from 30.5 to 14.5% was observed (Fig. S3a).

On a finer scale, the 16S rRNA sequences of the seeding sludge and the later sludge samples were assigned to 30 classes within 26 phyla (Fig. S3b). Similar to the phyla scale, the relative abundance of classes in the SrUASB reactor operated for 50 days did not change significantly (Fig. S3b). The fact that four out of ten reported SRB genera^39^ within *Deltaproteobacteria* were present in the sludge sample also indicated that sludge in the SrUASB reactor had a high diversity of the SRB community (Table 2 and Fig. S3c).

The *Desulfomonile* sp. was dominant in the seeding sludge, but after feeding of Hg(II) and Pb(II) for 50 days in the reactor, the *Desulfobacterium* sp. became dominant (see Table 2). *Desulfobacterium* is commonly identified in acid mine drainage treatment systems^40^,^41^,^42^. Buisman *et al.* also found that the *Desulfobacterium* sp. was able to reduce Hg(II) to a lower valence (elemental Hg)^43^, which is one of the mechanisms involved in the microbial detoxification of Hg salts^24^. This mechanism could explain the reason that feeding Pb(II) and Hg(II) for 50 days results in a predominance of the *Desulfobacterium* genus.

In addition to SRB, the fermentation bacteria (e.g., *Lactococcus*) almost disappeared after it was exposed to Pb(II) and Hg(II) for 50 days. However, *Trichococcus* became the dominant genus after the long-term feeding of Pb(II) and Hg(II), accounting for 12.8% of the bacterial community. The *Trichococcus* sp. plays an important role in the reduction of organic matters, particularly in decomposing complex organic matters into acetate, formate, lactate, etc., to facilitate the distinct reduction of sulfate/sulfite^44^,^45^. The dominance of *Trichococcus* sp. implies that the *Trichococcus* sp. may have a higher tolerance to Hg(II) and Pb(II) than the *Lactococcus* sp.

## Conclusions

In this study, long-term tests were conducted under low loading conditions of mercury and lead. In addition, short-term tests were conducted under high metal loading conditions. The aim of the study was to investigate the efficacy of Pb(II) and Hg(II) removal from synthetic FGD wastewater in a sulfite-reducing UASB reactor and in batch tests. During the tests, the impacts of mercury and lead on the activity, viability, and community of SRB were analyzed. The main findings are summarized as follows:

- The laboratory-scale SrUASB reactor performed satisfactorily in the 50-day tests at a Pb(II) loading rate of 9.2 g/m^3^-d and Hg(II) loading rate of 2.6 g/m^3^-d. At these loading rates, 72.5 ± 7% of SO_3_^2−^ and 86.4 ± 5% of COD were removed from the influent wastewater, although sulfite reduction generated 259 ± 30 mg/L sulfide on average. Meanwhile, both the influent Pb(II) and Hg(II) were effectively removed.
- Most of the removed Pb(II) and Hg(II) transformed into PbS and HgS, respectively. The contribution of cell adsorption and organic binding to Pb(II) and Hg(II) removal were only 20.0 ± 0.1% and 1.8 ± 1.0%, respectively. The different bioavailable concentration levels between lead and mercury resulted in different levels of lethal toxicity. As a result, a higher proportion of dead cells was observed to be induced by Pb(II) exposure than Hg(II) exposure.
- Given that the performance of the SrUASB reactor was stable and slightly increased in the long-term tests, the impacts of Hg(II) and Pb(II) on SRB activity were insignificant under the low metal loading conditions. In addition, 0.29 ± 0.21 μg MeHg per gram dry biomass was detected in the sludge of the SrUASB reactor.
- When the batch tests were exposed to high concentrations of Pb(II) or Hg(II) of 15–100 mg/L, the removal efficiencies of lead or mercury were constantly higher than 99.7%. The exposure to both Pb(II) or Hg(II) at the concentration of 100 mg/L increased the number of dead cells, but the increasing Hg(II) concentration showed a stimulatory effect on the sulfite reduction rate.
- This study confirms that the biological treatment for FGD wastewater via SRB under anaerobic conditions can effectively remove sulfite and organic matters as well as Pb(II) and Hg(II). Under low loading rates of Pb(II) and Hg(II), the operation of the SrUASB reactor was stable for a significantly long time, and the impacts of heavy metals were negligible.

## Materials and Methods

### Laboratory-scale SrUASB reactor operation

A laboratory-scale SrUASB reactor for the laboratory-scale flue gas desulfurization-sulfite reduction process, and an autotrophic denitrification and nitrification integrated (FGD-SANI) system^9^ were used in this work, as shown in Fig. S4. The inner diameter and height of the SrUASB reactor were 45 mm and 750 mm, respectively, resulting in 1.08 L of working volume. The seeding sludge was obtained from the Sha Tin Sewage Treatment Works in Hong Kong because it is rich in SRB. The practice of seawater toilet flushing brings 600–1000 mg/L sulfate into the sewage^46^,^47^,^48^. The initial sludge concentration was 20.0 g TSS/L (total suspended solids) or 15.5 g VSS/L. Detailed information regarding the SrUASB can be found in our previous work^9^.

In a biological FGD process, such as the Bio-FGD process initiated by Paque^12^, an inexpensive organic carbon source can provide the electron donor for the sulfite reduction. Organic matter in domestic wastewater is a suitable electron donor for SRB to reduce sulfite from the FGD wastewater. Therefore, the FGD wastewater was mixed with domestic wastewater to remove sulfite and organic carbon, as was proposed in the previous study of the FGD-SANI process^9^. The compositions of stock and trace element solutions are shown in Table S4. In addition, 1000 m g/L of stock solutions of mercuric chloride (HgCl_2_) and lead nitrate (Pb(NO_3_)_2_) were prepared. The influent concentration levels of Pb(II) and Hg(II) in the synthetic FGD wastewater (see below) were determined according to the U.S. EPA report^6^. The metal ions (Pb(II) and Hg(II)) contained in the synthetic FGD wastewater were mixed with synthetic domestic wastewater (1:5 in volume) and then pumped into the SrUASB reactor^9^. Thus, the influent of the SrUASB reactor consisted of 529 ± 50 mg/L COD, 386 ± 15 mg/L SO_3_^2−^S, 32 mg/L NH_4_^+^-N, 2.3 ± 0.4 mg/L Pb(II), and 570 μg/L Hg(II). Hydrochloric acid (HCl) of 1 mol/L was added to maintain the influent pH value at 7.1 ± 0.15 in the SrUASB reactor. The hydraulic retention time (HRT) and internal up-flow velocity were 5.2 h and 0.13 m/h, respectively. These findings were similar to our previous study of the FGD-SANI system^9^.

### Deposited mercury and lead compositions in the sludge

To investigate the mechanisms of Pb(II) or Hg(II) removal during the sulfite-reducing process, the mercury deposited in the sludge and the composition of lead in the sludge were analyzed. Sludge taken from the SrUASB reactor (2.5 g VSS/L) was washed with deionized water three times and then evenly distributed into two 100 ml flasks. Afterwards, the mixed synthetic FGD-domestic wastewater was added, using the procedure established for the SrUASB reactor. To stimulate the total amount of influent Pb(II) and Hg(II) in the SrUASB reactor daily, concentrated solutions of Pb(II) and Hg(II) were spiked into the flasks at the loading rates of 9.2 g/m^3^-d Pb(II) and 2.6 g/m^3^-d, respectively. Metal-free controls were also performed, as were duplicate tests. After 48 hours, the sludge in each flask was removed and analyzed. Lead and mercury deposited in the sludge sample were extracted following a five-step sequential leaching-extraction operation^49^. Afterwards, the exchangeable, organically bound, carbonate-bound, Fe/Mn oxides-bound and sulfide-bound metals were determined.

### Batch tests of toxicity of Pb(II) and Hg(II) to SRB

Batch tests were conducted to evaluate the activity of SRB, such as SRR, and bacterial viability under extremely high metal concentrations. Different concentrations of Pb(NO_3_)_2_ and HgCl_2_ in the mixed FGD-domestic wastewater showed sharp increases.

Fourteen sets of batch tests were conducted. The wastewater was first fed with Pb(II) and/or Hg(II) to simulate the individual Pb(II) feeding concentrations of 0, 15, 30, 75, and 100 mg/L. Then, individual Hg(II) feeding concentrations were set at 0, 15, 30, 75 and 100 mg/L; and mixed Pb(II) and Hg(II) feeding concentrations were set at (a) 30 mg/L Pb(II) and 30 mg/L Hg(II), (b) 30 mg/L Pb(II) and 100 mg/L Hg(II), (c) 100 mg/L Pb(II) and 30 mg/L Hg(II), and (d) 100 mg/L Pb(II) and 100 mg/L Hg(II). The tests were run in parallel for 96 hours using 500 ml flasks. Sludge taken from the laboratory-scale SrUASB reactor was added to each flask as 1.1 ± 0.1 g/L mixed-liquid VSS. The composition of the mixed synthetic FGD-domestic wastewater was similar to the laboratory-scale test, except for the concentrations of Pb(II) and Hg(II). All sealed flasks were continuously magnetically stirred at 400 rpm and kept at ~25 °C. Approximately 60% of the supernatant in each flask was renewed every 24 hours after settling for 30 minutes, and the Pb(NO_3_)_2_ and HgCl_2_ were intentionally added again to the initial concentrations as above. Thus, the actual loading rates were kept between 15–100 g/m^3^-d (see Table 1) for the batch tests. Samples were taken from the supernatant every 24 hours to analyze the changes of Hg(II), Pb(II), organic matter, and sulfide concentrations and to determine the SRR.

### Cell Viability

After the batch tests, three sludge samples in the 100 mg/L Pb(II)-fed flask, 100 mg/L Hg(II)-fed flask, and metal-free control flask were taken to evaluate the bacterial viability. The live and dead fractions of the sludge samples exposed to Pb(II) or Hg(II) for 96 h were measured using a bacterial viability assay^50^.

Samples were stained according to the instructions provided with the BacLight LIVE/DEAD^TM^ counting Kit (L34856, Invitrogen, Eugene, OR, USA), which employs a combination of green fluorescent SYTO 9 dye and red fluorescent propidium iodide (PI) for viability assessment. Live/Dead cell quantification was performed using a FC500 flow cytometer (Beckman Coulter, Brea California, USA) equipped with a 15 mW argon ion laser, emitting at a fixed wavelength of 488 nm. This instrument employs a solid-state photodiode detector for the forward scatter signal and photo multiplier tubes to quantify the fluorescence and side scatter signals. Fluorescence was recorded at the logarithmic signal amplification. Data were collected using the Cell Quest software (Beckman Coulter). The samples were placed in 12 × 75 nm plastic tubes.

### Analytical methods

Influent and effluent samples were collected daily and analyzed after filtration (Millipore, 0.45 μm). These included TOC, dissolved sulfide (H_2_S, HS^−^ and S^2−^), sulfite, thiosulfate, Pb(II) and Hg(II). To eliminate the effect of dissolved sulfide on COD measurement, TOC was determined by a TOC Analyzer (Shimadzu TOC-5000A) and then converted into COD according to the ratio of COD to TOC (2.67)^51^. Dissolved sulfide, total suspended solids (TSS), and volatile suspended solids (VSS) were measured according to standard methods^52^. In the SrUASB reactor, Pb(II) was analyzed using a Z-5000 flame atomic adsorption spectrometer (FAAS) (Hitachi, Japan) with detection limits of 0.01 mg/L. However, Pb(II) was quantified in the batch tests using graphite furnace atomic adsorption spectrometry (AAS ZEEint-60) with detection limits of 1.1 μg/L. Hg(II) was measured using an atomic fluorescence spectrometer with detection limits of 0.001 μg/L (AFS-820, Beijing Jitian Instrument). A calibration curve was prepared by measuring the absorbance of a series of standard solutions of known Hg(II) concentrations. No mercury was found in the sample blanks (MQ water only). To meet the limits, the influent and effluent water samples were diluted with 5% HNO_3_ solution containing 0.5 g/L K_2_Cr_2_O_7_ before analysis. Sulfite and thiosulfate were analyzed using an ion chromatograph (DIONEX-900) after filtration. In the batch tests, all samples were analyzed immediately after filtration by disposable Millipore filters (0.45 μm pore size). The pH was measured by a pH meter (HQ40D).

The MeHg in the sludge sample was extracted and then analyzed by high-speed liquid chromatography (Agilent 1260) and inductively coupled plasma mass spectrometry (Thermo Fisher Scientific iCAP^TM^ Q, HPLC-ICP-MS) in time-resolved analysis (TRA) mode. The optimized HPLC-ICP-MS operating conditions are summarized in the Supporting Information. The extraction protocol was based on the method established by Chen *et al*.^53^ A sludge sample of 3 mL was collected from the reactor and pipetted into a 50 mL polyethylene centrifuge tube. Then, 6 mL of 5 mol/L HCl were added. The tube was kept in an ultrasonic bath for 30 min at room temperature. Afterwards, the tube was centrifuged for 4000 rpm for approximately 10 min. and the supernatant was collected. Triple extractions were made. All extracted supernatants were combined, then diluted into a 100 mL volumetric flask with ultra-pure water and filtered through a 0.45 μm pore-size membrane prior to analysis.

Mercury and lead deposited in the sludge were extracted and separated into the following categories: (i) exchangeable, (ii) bound to carbonates, (iii) bound to Fe/Mn oxides, (iv) bound to organic matter, and (v) residues. This analysis was performed using the Tessier sequential extraction procedure described by Akcay *et al*.^49^ The speciations of mercury and lead were then analyzed using an atomic fluorescence spectrometer (AFS-820).

### Microbial community analysis

#### DNA extraction, PCR amplification, pyrosequencing

DNA was extracted from the sludge samples of the SrUASB reactor collected on days 1 and 50 by using a fast DNA SPIN Kit for soil (Qbiogene Inc). A combination of tagged primers designed for the variable ITS-1 region was applied to amplicon libraries according to the tag-encodes 454 GS-FLX amplicon pyrosequencing method^54^. The method of fragments for the 16S rRNA amplification was used as previously described^9^. The broad-specificity primers were designed by adding a 6-nucleotide barcode (5′-ACACATAT-3′) to the universal forward primer 515F (5′-CCATCTCATCCCTGCGTGTCTCCGACTCAGACACATATGTGCCAGCMGCCGCGGTAA-3′) and the reverse primer 926R (5′-CCTATCCCCTGTGTGCCTTGGCAGTCTCAGCCGTCAATTYYTTTRAGTTT-3′) for amplification of bacteria^55^. The detailed PCR conditions were adopted as described by Lee *et al*.^56^, using a Wizard^®^ SV Gel and PCR Clean-up System (Promega, Madison, Wisconsin, USA) to purify the equimolar amounts of amplicons. The purified 16s rRNA amplicons were subjected to pyrosequencing with a ROCHE 454 FLX Titanium platform (Roche, Basel, Switzerland) at the National Human Genome Center of China at Shanghai, China (CHGC). Tag sequences were then selected according to the sequence criteria^57^.

#### Sequence analysis

After trimming the low quality sequences, residual sequences were aligned using the EMBOSS package^58^. Operational taxonomic units (OTUs) were then obtained at 97% similarity using BLASTclust v.2.2.1.6^59^. The calculated OTUs were applied to the analyzed rarefaction curves, richness (Shannon) and diversity indices (Chao1) using MOTHUR ver. 1.17.0^60^. The bacterial community from genus to phylum was then obtained using the RDP classifier (Ribosomal Database Project (RDP)^61^, the National Center for Biotechnology Information (NCBI), BLAST^62^, and the Greengenes databases^63^.

## Additional Information

**How to cite this article**: Zhang, L. *et al.* Effects of Lead and Mercury on Sulfate-Reducing Bacterial Activity in a Biological Process for Flue Gas Desulfurization Wastewater Treatment. *Sci. Rep.* **6**, 30455; doi: 10.1038/srep30455 (2016).

## Supplementary Material

Supplementary Information

## Figures and Tables

**Table 1:** Characteristics of the influent and effluent wastewater of the SrUASB reactor and comparisons of the main performance with SANI and FGD-SANI processes.

Processes	Parameters	Influent	Effluent	Removal efficiency
This study	COD (mg/L)	529 ± 50	72 ± 35	86.4 ± 5%
	SO_3_^2−^-S (mg S/L)	386 ± 15	106 ± 30	72.5 ± 7%
	Dissolved sulfide (mg S/L)	0	259 ± 30	—
	S_2_O_3_^2−^-S (mg S/L)	—	26 ± 20	—
	pH	7.10 ± 0.15	7.41 ± 0.21	—
	Pb(II) (mg/L)	2.3 ± 0.4	<0.01*	>99.5%
	Hg(II) (μg/L)	570	1.1 ± 0.9	99.8 ± 0.1%
Sulfate-reducing UASB (SANI^25^)	COD (mg/L)	273 ± 13	30.7 ± 1.2	88.7 ± 4%
	SO_4_^2−^-S (mg S/L)	185 ± 8	78 ± 4	57.8 ± 3%
	Dissolved sulfide (mg S/L)	—	88.0 ± 4.1	—
Sulfite-reducing UASB (FGD-SANI^10^)	COD (mg/L)	529 ± 13	69 ± 5	86.9%
	SO_3_^2−^-S (mg S/L)	386 ± 10	130 ± 3	66.3 ± 2%
	Dissolved sulfide (mg S/L)	—	247 ± 10	—

*The measured values were all below the detection limits.

**Table 2:** Relative abundance of sulfite-reducing and fermentation-related genera.

Sludge samples		G1 (day 1) (%)	G2 (day 50) (%)
Sulfite-reducing genera	*Desulfomonile*	9.1	3.1
	*Sulfurovum*	0.5	0.5
	*Desulfobulbus*	0.1	0.1
	*Desulfobacterium*	0	6.9
Fermentation-related genera	*Trichococcus*	14.2	12.8
	*Lactococcus*	8.6	0.1
