# Supplementary material for: Effects of Lead and Mercury on Sulfate-Reducing Bacterial Activity in a Biological Process for Flue Gas Desulfurization Wastewater Treatment
Source: Sci Rep. 2016 Jul 26;6:30455. doi: 10.1038/srep30455 (PMC4960525; doi:10.1038/srep30455)
Supplement: Supplementary Information [file srep30455-s1.doc]

**Supplementary Information**

**Effects of Lead and Mercury on Sulfate-Reducing Bacterial Activity in a Biological Process for Flue Gas Desulfurization Wastewater Treatment**

Liang Zhanga, Xiaojuan Lina,c, Jinting Wanga, Feng Jianga, b,*, Li Weid­, Guanghao Chend, Xiaodi Haoe

*aSchool of Chemistry & Environment, South China Normal University, Guangzhou, China*

*bKey Laboratory of Theoretical Chemistry of Environment, Ministry of Education, Guangzhou, China*

*cSYSU-HKUST Research Center for innovation Environmental Technology, Sun Yat-sen University, Guangzhou, China*

*dDepartment of Civil & Environmental Engineering, The Hong Kong University of Science and Technology, Clear Water, Kowloon, Hong Kong, China*

*eKey Laboratory of Urban Stormwater System and Water Environment -MoU/R and D Centre for Sustainable Wastewater Treatment, Beijing University of Civil Engineering and Architecture, Beijing, China*

**corresponding author:* Feng Jiang

Address: Waihuan Rd. West, Higher Education Mega Center, Guangzhou, China

Email:[**jiangfeng@scnu.edu.cn**](mailto:jiangfeng@scnu.edu.cn)

Tel: +86-20-39310353

Fax: +86-20-39310187

**Determination method of MeHg**

MeHg in the sludge sample was extracted and then analyzed by high performance liquid chromatography (Agilent 1260)-inductively coupled plasma mass spectrometry (Thermo Fisher Scientific iCAPTM Q)(HPLC-ICP-MS) in a time resolved analysis(TRA) mode. The optimized HPLC-ICP-MS operating conditions were summarized in the following Table S1. The spectrum of the HPLC-ICP-MS analysis was proposed in Figure S1.

| **Table S1.** Optimized operational conditions for the HPLC-IC-MS system | |
| --- | --- |
| **ICP-MS parameters** | |
| RF power | 1550W |
| Cool gas flow rate | 14L/min |
| Auxiliary gas flow rate | 0.8L/min |
| Neubilizer gas flow rate | 0.88L/min |
| **HPLC system** | |
| Mobile phase | 60mM ammonium acetate, 5%(v/v)methanol,  0.1%(v/v)2-mercaptoethanol |
| Flow rate of the mobile phase | 0.4mL/min |


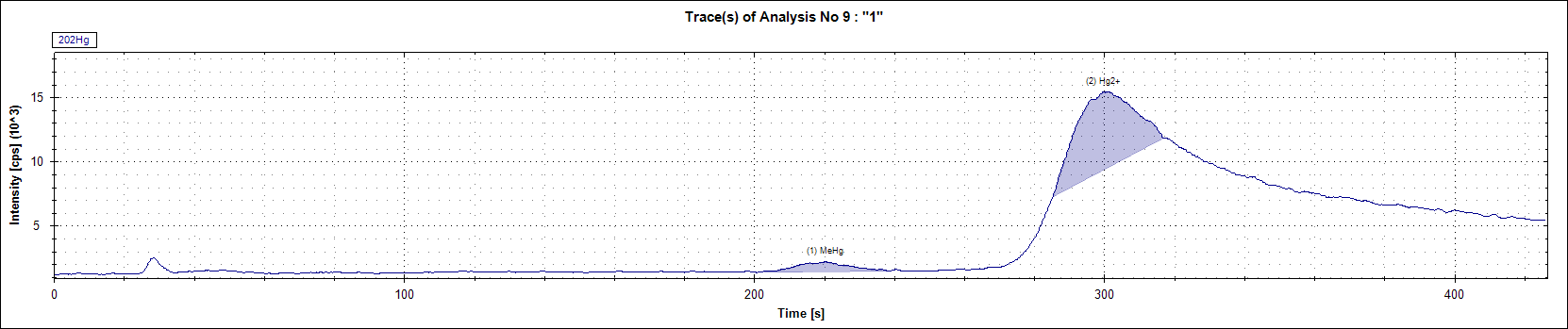


Figure S1. Spectrum of HPLC-ICP-MS to detect methylmercury in the sludge samples.

**Sequential extraction procedure for the speciation of mercury and lead in sludge sample**

The speciation of mercury and lead was extracted using the Tessier sequential extraction procedure, described below.

(i) Exchangeable:

All of the sludge after the batch test was collected in a 100 mL conical flask. Then, the sludge was extracted at room temperature for 1 h with 10 mL of magnesium chloride solution (1 M MgCl2 buffered at pH 7.0) with continuous agitation. The supernatant was collected by centrifugation at 3000 rpm for approximately 30 min.

(ii) Bound to Carbonates:

The residue from (i) was leached at room temperature with 10 mL of 1 M NaOAc adjusted to pH 5.0 with acetic acid (HOAc). Continuous agitation was maintained for 5 h. The supernatant was collected by centrifugation at 3000 rpm for approximately 30 min.

(iii) Bound to Fe-Mn Oxides:

The residue from (ii) was extracted with 20 mL 0.04 M NH2OH·HCl in a 25% (v/v) HAc. The latter experiments were performed for 6 h at 96±3℃ with occasional agitation. The supernatant was collected by centrifugation at 3000 rpm for approximately 30 min.

(iv) Bound to Organic Matter:

To the residue from (iii) was added 3 mL of 0.02 M HNO3 and 5 mL of a 30% H2O2, adjusted to pH 2 with HNO3, and the mixture was heated to 85±2℃ for 2 h with occasional agitation. A second 3 mL aliquot of 30% H2O2 (PH 2.0 with HNO3) was then added, and the sample was heated again to 85±2℃ for 3 h with intermittent agitation. After cooling, 5 mL of 3.2 M NH4OAc in a 20% (v/v) HNO3 was added and agitated continuously for 30 min. The supernatant was collected by centrifugation at 3000 rpm for approximately 30 min.

(v) Residues:

The residue from (iv) was digested for 3 h with the mixture of 8.0 mL of HNO3, 2.0 mL of HF, 2.0 mL of HClO4 and 2.0 mL of HCl. The digestion was performed at 85±2℃ with occasional agitation. The supernatant was collected by centrifugation at 3000 rpm for approximately 30 min.

The supernatant of each step was collected separately for analysis using an atomic fluorescence spectrometer (AFS-820).


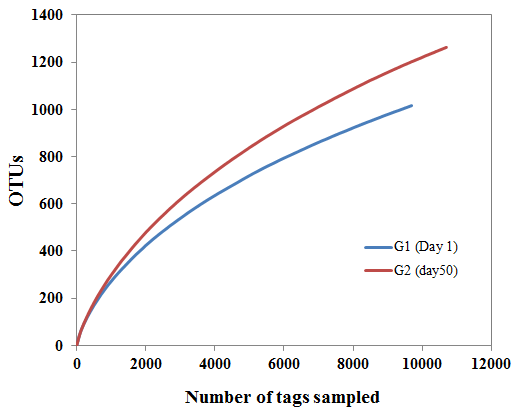


**Figure S2**. Rarefaction analysis of the sludge sample from the SrUASB reactor. Rarefaction is shown for OTUs with differences that do not exceed 3% OTUs with ≥97% pairwise sequence identity are assumed to form the same species and genus, respectively.

### Figure S3. Taxonomic classification of bacterial 16S rRNA gene reads of the sludge sample from the SrUASB reactor at phylum (a), class (b) and genus (c) levels (the relative abundances of bacterial 16S rRNA gene reads below 0.1% are not shown). G1-seeding sludge, G2-sludge of day 50.


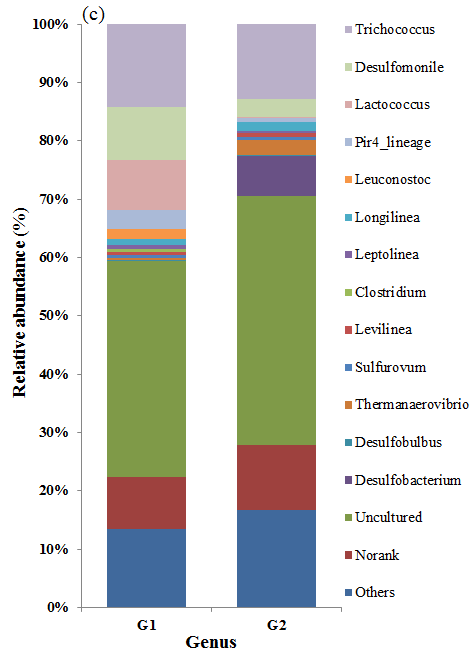

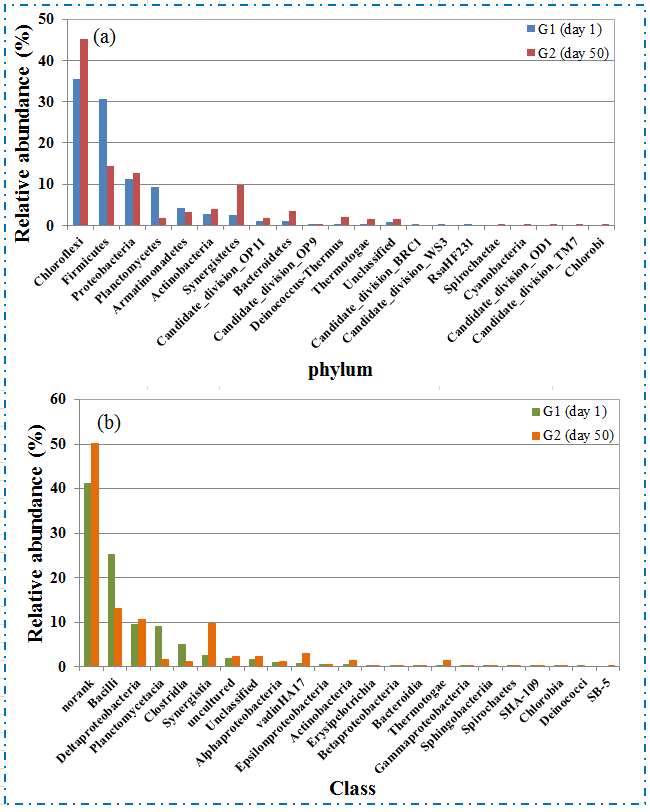


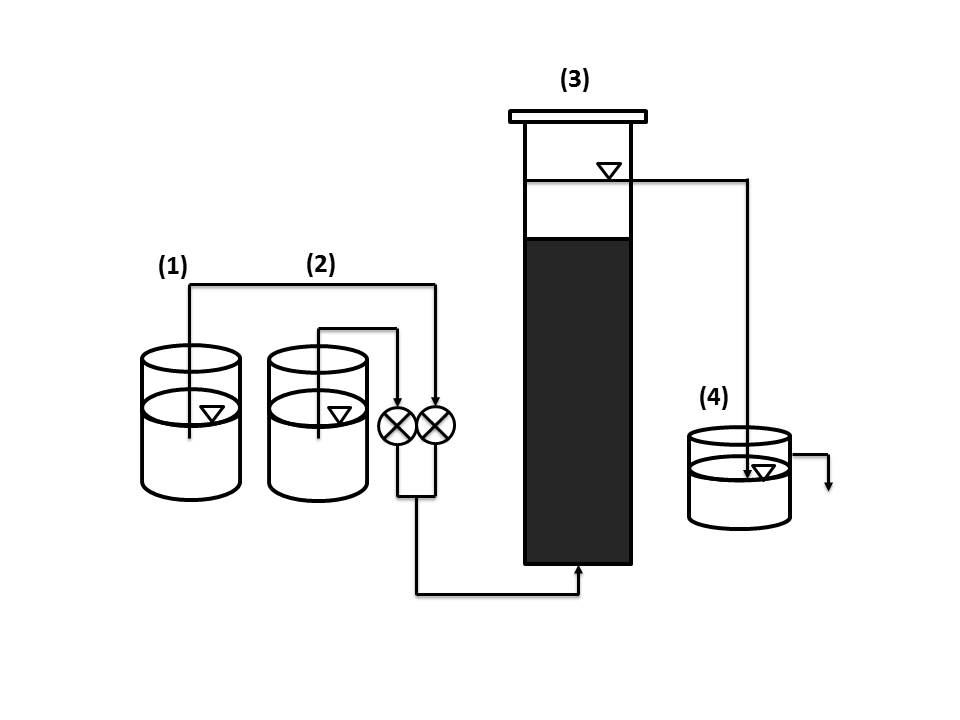


**Figure S4**. Schematic diagram of SrUASB reactor. (1) Pb(II) and Hg(II) containing sodium sulfite solution, (2) Synthetic domestic wastewater, (3) SrUASB reactor, (4) Effluent tank.

| **Table S2**. Average effluent concentrations and removal efficiency of lead and mercury under different concentrations in the batch tests. | | | | | | | | | | |
| --- | --- | --- | --- | --- | --- | --- | --- | --- | --- | --- |
| **Individual feeding of Pb**(**II) or Hg(II)** | | | | | | **Mixed feeding of Pb(II)and Hg(II)** | | | | |
| **Pb**(**II)** | | | **Hg**(**II)** | | |  | | | | |
| Influent loading rate (g/m3-d) | Effluent concentration (µg/L) | Removal efficiency (%) | Influent loading rate (g/m3-d) | Effluent concentration (µg/L) | Removal efficiency (%) | Influent loading rate Pb(II)+Hg(II) (g/m3-d) | Effluent concentration (µg Pb/L) | Effluent concentration (µg Hg/L) | Removal efficiency ( Pb, %) | Removal efficiency (Hg, %) |
| 15 | 6.9 | 99.953 | 15 | 40.1 | 99.722 | 30+30 | 0.8 | 19.1 | 99.997 | 99.940 |
| 30 | 17.2 | 99.941 | 30 | 36.5 | 99.885 | 30+100 | 4.4 | 11.5 | 99.996 | 99.964 |
| 75 | 11.5 | 99.980 | 75 | 69.1 | 99.904 | 100+30 | 9.1 | 17.0 | 99.969 | 99.983 |
| 100 | 20.2 | 99.980 | 100 | 48.7 | 99.952 | 100+100 | 3.6 | 7.0 | 99.998 | 99.993 |

| **Table S3.** The number of OTUs and species richness estimates with a confidence threshold of 97% obtained from the sludge samples G1 and G2 respectively. | | | | | | |
| --- | --- | --- | --- | --- | --- | --- |
| Sample | OTUs | ace | chao | shannon | simpson | coverage (%) |
| G1 (day 1) | 10182 | 2336.382 | 1776.506 | 4.731981 | 0.032519 | 99.97583 |
| G2 (day 50) | 11862 | 2913.537 | 2277.134 | 4.770464 | 0.041381 | 99.96995 |

| Table S4. Composition of stock and trace element solutions. | | | |
| --- | --- | --- | --- |
| **Stock solution** | | | |
| Component | Concentration (g/L) | Component | Concentration (g/L) |
| Sodium acetate | 15.38 | NH4Cl | 5.42 |
| Glucose | 11.51 | K2HPO4 | 1.32 |
| Yeast extract | 5.76 | KH2PO4 | 0.426 |
| **Stock trace element solution** | | | |
| Component | Concentration (g/L) | Component | Concentration (mg/L) |
| FeCl3·6H2O | 2 | ZnSO4·7H2O | 0.15 |
| MnSO4·4H2O | 0.25 | KI | 0.08 |
| CoCl2·6H2O | 0.2 | CuSO4 | 0.05 |
| H3BO3 | 0.2 |  |  |
